# Draft Genome Sequence of Methicillin-Resistant Staphylococcus aureus Strain AW7, Isolated from a Patient with Bacteremia

**DOI:** 10.1128/MRA.00806-19

**Published:** 2019-10-03

**Authors:** David R. Cameron, Alban Ramette, Josef Prazak, José Entenza, Matthias Haenggi, Yok-Ai Que, Grégory Resch

**Affiliations:** a Department of Intensive Care Medicine, Inselspital, Bern University Hospital, University of Bern, Bern, Switzerland; b Institute for Infectious Diseases, University of Bern, Bern, Switzerland; c Department of Fundamental Microbiology, University of Lausanne, Lausanne, Switzerland; University of Rochester School of Medicine and Dentistry

## Abstract

Methicillin-resistant Staphylococcus aureus (MRSA) strain AW7 is a commonly used challenge strain in experimental models of MRSA infection. Here, we report its draft genome sequence.

## ANNOUNCEMENT

Methicillin-resistant Staphylococcus aureus (MRSA) strain AW7 (complete taxonomy is *Bacteria*, *Firmicutes*, *Bacilli*, *Bacillales*, *Staphylococcaceae*, Staphylococcus aureus strain AW7) was isolated from a patient with bacteremia in Switzerland in the 1980s. The strain has been used for many decades in experimental endocarditis models aimed at testing antibiotic efficacy (1, 2). More recently, we have used AW7 in a rodent model of ventilator-associated pneumonia to assess phage therapy (3).

AW7 was grown overnight at 37°C in tryptic soy broth. Genomic DNA was prepared using a Qiagen blood and tissue kit per the manufacturer’s instructions and sequenced using the Illumina MiSeq platform (Nextera DNA Flex; assay V2 2 × 150 bp, 300 cycles), generating 6,274,783 reads. Sequencing data were processed using the shovill pipeline with the –trim option and default parameters (v0.9.0). The average read length was 148 bp with an avgQscore of 37.6. Contigs were assembled using SPAdes (v3.11.1; –only-assembler; k-mer sizes of 31, 51, 71, 91, and 111) (4) and polished using Pilon (v1.22) (5). The sequence comprised 63 contigs (>200 bp long; *N*_50_, 140,137 bp) with a combined length of 2,910,678 bp (G+C content of 32.7%) and an average coverage of ∼62×. The draft genome was annotated using the NCBI Prokaryotic Genome Annotation Pipeline (v4.8) (6), resulting in 2,918 predicted protein-coding regions.

AW7 belongs to multilocus sequence type 247 (ST247) (7) and harbors a staphylococcal cassette chromosome *mec* type I (1B) [SCC*mec* I (1B)] element (as determined by SCC*mec* finder v2.1). In addition to SCC*mec*, which facilitates methicillin resistance, AW7 harbors genes predicted to code for additional resistance determinants for β-lactams (*blaZ*), aminoglycosides [*aph(3′)-III*, *ant(6)-Ia*, *ant(9)-Ia*, and *aac(6′)-aph(2″)*], tetracycline (*tetM*), macrolide-lincosamide-streptogramin B (*ermA*) (2), and chloramphenicol (*cat*) (8). The genome contains three prophages, as determined by PHAST (9), including a β-hemolysin-converting phage that harbors virulence factors enterotoxin A (*sea*), staphylokinase (*sak*), and complement inhibitor (*scn*). Additional virulence factors include toxin genes α-hemolysin (*hla*), γ-hemolysin (*hlgABC*), and leukocidin (*lukDE*), as well as exoenzymes aureolysin (*aur*), serine protease A (*splA*), serine protease B (*splB*), and serine protease E (*splE*) (10).

The genome sequence of AW7 provides a useful resource for future studies of MRSA infection in experimental animal models.

### Data availability.

This whole-genome shotgun project has been deposited at DDBJ/ENA/GenBank under the accession number SRLL00000000, with SRA accession number PRJNA530527.
